# “Availability is the poor cousin of marketing and pricing”: qualitative study of stakeholders’ views on policy priorities around tobacco and alcohol availability

**DOI:** 10.1080/09687637.2023.2282355

**Published:** 2023-11-25

**Authors:** Elena D. Dimova, Niamh K. Shortt, Richard J. Mitchell, Peter Lekkas, Jamie R. Pearce, Tom L. Clemens, Carol Emslie

**Affiliations:** a Glasgow Caledonian University, Glasgow, Scotland, UK; b Centre for Research on Environment, Society and Health, School of GeoSciences, University of Edinburgh, Edinburgh, UK; c MRC/CSO Social and Public Health Sciences Unit, Institute for Health and Wellbeing, University of Glasgow, Glasgow, UK

**Keywords:** Alcohol, tobacco, availability, policy, public health

## Abstract

**Background:**

Reducing alcohol and tobacco availability is one potential way to reduce harm from these unhealthy commodities. This study explores key stakeholders’ views in relation to policy priorities and considerations for both alcohol and tobacco availability.

**Methods:**

We conducted semi-structured interviews with 14 stakeholders from alcohol and/or tobacco third sector organizations, government, public health and licensing in Scotland. Interviews explored their views on alcohol/tobacco availability, including its place in the policy landscape and experiences in gaining support for policies. Data were analyzed using thematic analysis.

**Results:**

Stakeholders believed that alcohol and tobacco availability have not received as much policy attention as pricing and marketing. Stakeholders highlighted the importance of public support and having sufficient evidence to inform policy. Key considerations for future policies include: drawing on lessons from tobacco control policies to address alcohol availability, considering different aspects of availability (especially online availability), ensuring policies reflect their local context, considering the impact of policies on children, and managing retailers’ involvement in the policy process.

**Conclusion:**

This study highlights key considerations for policies to address alcohol and tobacco availability. There is a need for more research to consider retailers’ views and provide greater detail on specific policy suggestions.

## Introduction

Tobacco and alcohol use are major determinants of preventable morbidity and mortality. Globally, seven million lives are lost each year to tobacco-related illness (WHO, 2021) and three million to alcohol-related illness (WHO, 2018). Scotland has the highest rate of alcohol-specific deaths and highest prevalence of cigarette smoking in the UK (ScotPHO, 2022; NRS, 2022). Recent data show over 9300 smoking-related deaths (ScotPHO, 2022) and over 1,200 alcohol-specific deaths (NRS, 2022) per year. Alcohol and tobacco consumption are key factors in driving the rise in health inequalities seen in most high income countries in recent decades; both can be mediators between social disadvantage and poor health (Loring 2014a, 2014b). Population-level policies to address alcohol and tobacco consumption have the potential to reduce premature mortality and morbidity, and health inequalities (Martineau et al., 2013; Thomas et al., 2008).

The local environment is an important determinant of smoking and alcohol related behaviors, including the local marketing, pricing and availability of unhealthy commodities. The focus here is the local availability of alcohol and tobacco products. High alcohol and tobacco availability are associated with increased alcohol and tobacco-related harms, especially in areas of multiple deprivation (Macdonald et al., 2018; Pearce et al., 2020; Richardson et al., 2015; Shortt et al., 2015). Literature suggests that greater neighborhood availability of tobacco and alcohol retail outlets increases supply of these products; raises awareness of tobacco/alcohol brands; creates competitive local markets that reduce product costs; and affects local social norms relating to consumption (see Clemens et al., 2018; Pearce et al., 2012; Shortt et al., 2016 for discussion of these possible pathways).

Addressing the supply and availability of tobacco products has been identified as the next frontier in this area (Cohen & Anglin, 2009). The World Health Organisation (2019) recommends restriction of alcohol and tobacco availability as a cost-effective way to prevent non-communicable diseases. Systematic review evidence demonstrates promising effects of availability interventions on reducing smoking prevalence and alcohol use (Campbell et al., 2009; Martineau et al., 2013; Thomas et al., 2008; Wilson et al., 2012). Effective interventions have addressed spatial (e.g., exclusion zones around schools; reduced number of retail outlets) and temporal (e.g., restricting hours of sales) availability (Lee et al., 2022; Martineau et al., 2013). Smoking and alcohol consumption can be concurrent behaviors (Aekplakorn et al., 2008; Room, 2004), and approaches to target them can be similar (WHO, 2017). For example, a reduction in the demand of both goods could be achieved by raising the price of just one of them (Pierani & Tiezzi, 2009; Young-Wolff et al., 2014). Smoke-free policies have been shown to reduce heavy drinking among people smoking in pubs and bars (McKeet et al., 2009) and may lead to reduction in overall alcohol consumption per capita, especially when implemented alongside pricing policies (Krauss et al., 2014). Additionally, comprehensive smoke-free regulations did not lead to increased drinking at home among smokers in Ireland (Hyland et al., 2008). High availability and easy access to alcohol have been linked to higher rates of smoking among US students (Weitzman et al., 2005). Although there is a need for greater knowledge on potential synergistic effect of policies addressing both behaviors (Gillespie et al., 2021), alcohol policy advocates may seek to learn from the success of tobacco control policies, and vice versa.

Implementation of public health control policies is complex, requires collaboration between various stakeholders and is influenced by social and cultural factors within and outside of organizations (WHO, 2022). Factors such as political commitment and collaboration with the voluntary sector can promote policy implementation (Holden & Hawkins, 2012; Wu et al., 2018). A recent scoping review of barriers and facilitators to the implementation of effective alcohol control policies identified more barriers (e.g., lack of evidence; low policy priority; exploitation of legal loopholes) than facilitators (Jankhotkaew et al., 2022).

This paper aims to advance understanding of key priorities and considerations to addressing alcohol and tobacco availability. It is based on a study, conducted in Scotland, although the findings have international relevance. Scotland experiences wide inequalities in alcohol and tobacco-related harm. The Scottish Government has taken radical action to address these by reducing alcohol affordability (i.e., Alcohol Minimum Unit Pricing in 2012) and tobacco marketing and visibility (e.g., plain packaging; a ban on the open display of tobacco at the point of sale). However, existing strategies have had very limited focus on reducing alcohol and tobacco availability (Scottish Government, 2018a; 2018b). There are over 17, 000 places to buy alcohol and over 10,000 places to buy tobacco in Scotland (population 5.4 million) (Pearce et al., 2020; Shortt et al., 2015; Scotland’s Census, 2023).

This paper focuses on the views of key stakeholders, working in the fields of alcohol and tobacco, in relation to policy priorities and considerations for both alcohol and tobacco availability in Scotland. The interviews are part of a broader mixed-methods study of alcohol and tobacco availability in Scotland (Dimova et al., 2023; Shortt, 2019). The specific questions we aimed to answer were: 1) What is the place of availability (compared to affordability and marketing) in alcohol and tobacco policy in Scotland?; 2) What do stakeholders perceive to be the policy priorities in relation to alcohol and tobacco availability in Scotland?; 3) What policies have the potential to reduce inequalities?; 4) What are stakeholders’ experiences of leveraging support for policies?.

## Methods

This study involved qualitative semi-structured interviews with public health stakeholders from third sector organizations and alcohol licensing, and policy makers. Qualitative methods are appropriate for exploring the views and experiences of study participants and can identify emergent themes not considered at the research design stage (Petticrew et al., 2013).

Ethical approval was granted by the Nursing Department Research Ethics Committee at Glasgow Caledonian University (HLS/NCH/20/033, June 2021).

## Participants and recruitment

We used pragmatic purposive sampling and snowball referrals (Bryman, 2012). We recruited stakeholders who had interest and experience in tobacco and/or alcohol policies and were familiar with the policy landscape in Scotland. Potential participants were identified through professional networks, then emailed and invited to take part. The respondents (n = 14, ten women, four men) were from the following sectors: third sector, including organizations working in alcohol and/or tobacco, policy, public health and alcohol licensing. Time in current role varied from four months to 17 years.

We also invited retailers (supermarkets and convenience stores) to take part in the study but all declined or did not respond.

## Data collection

We conducted 11 interviews with 14 participants (10 one-to-one, one with four policy makers) between October 2021 and February 2022. Due to the COVID-19 pandemic, all interviews were conducted remotely via a video call. Interview duration varied from 40 to 82 minutes, with an average length of 57 minutes. A study information leaflet and consent form were given to participants and informed consent was recorded prior to the interview. To maintain anonymity, each respondent was assigned a unique ID number.

The interviews followed a semi-structured topic guide, which included open-ended questions about alcohol/tobacco availability in Scotland, including its place in the policy landscape compared to affordability and marketing, policy considerations in relation to inequalities and experiences in gaining support for policies (Supplementary Material 1).

## Data analysis

With permission, all interviews were audio recorded and then transcribed by professional transcribers. Transcripts were checked for accuracy by a member of the research team (ED), who also removed any identifying information.

Data were entered into NVivo v12, a software programme designed to aid analysis of qualitative data, and analyzed using thematic analysis (Braun & Clarke, 2006; Clarke & Braun, 2017). After initial familiarization with the data, a coding framework was developed by one researcher (ED) based on the interview schedule and discussed with CE. This initial coding framework was structured into broad categories: overall views on alcohol/tobacco availability in Scotland, experiences in gaining support for policies and involving retailers in policy discussions, and policies to reduce inequalities. The transcripts were then coded line-by-line by one researcher (ED) using a mixture of deductive coding based on the coding framework and an inductive, open-coding approach based on emerging issues. Following this, high level themes were identified individually by ED and CE, and discussed. Themes were then discussed with the whole team. The final themes reflect the study research questions and focus on the place of availability in the policy context; policy priorities for addressing availability; addressing alcohol and tobacco health inequalities; and policy support and conflict of interest.

## Findings

First, we focus on stakeholders’ views on the place of availability in the current policy landscape, and policy priorities for addressing alcohol and tobacco availability in Scotland. We then explore views on potential ways for policies to address inequalities. Finally, we present stakeholders’ experiences in leveraging support for policies and managing conflicts of interest.

### The place of availability in the policy context: “the poor cousin of marketing and pricing promotion”

There was a common perception among stakeholders that tobacco and alcohol are easily available in Scotland, and often bought from the same place:

*“There is just the sheer volume of locations that you can purchase alcohol or tobacco and usually they’re the same place”* (S2, Third sector organisation)

Stakeholders highlighted that availability is closely linked to pricing and marketing, especially in relation to alcohol (as marketing of tobacco products is banned in Scotland):

*“If you go slightly beyond availability and think about the availability of marketing, you don’t need to walk far before you get told that your nearest place to buy a bottle of wine’s round the corner.”* (S2, Third sector organisation)

*“One of the gaps is probably a lack of understanding of how availability influences exposure to marketing (…) sponsorship, promotion, availability has a really fundamental role to play in that.”* (S8, Third sector organisation)

Third sector participants believed that despite these *“inter-dependencies,”* alcohol and tobacco availability have not received as much policy attention within the Scottish Government as have pricing and marketing. One participant described availability as *“the poor cousin of marketing and pricing promotion”* (S2, Third sector organisation). They gave examples of policies to reduce tobacco marketing (e.g., standardized packaging; point of sale ban) but highlighted that tobacco is still *“easily available”* as *“you don’t need to go far to buy a packet of cigarettes”* (S2, Third sector organisation*):*

*“I do think for the scale of the damage it [tobacco] does, which is over nine thousand preventable deaths every year at this point [in Scotland], and a hundred thousand hospital admissions every year, it is way more available and visible than is good for us.”* (S5, Third sector organisation)

Some third sector stakeholders suggested that although alcohol and tobacco are both health-harming products, “*there are more significant gaps* [in health policies] *in alcohol than there are in tobacco”* (S3, Third sector organisation). To back this up, they listed policies in Scotland on pricing and taxation of tobacco, standardized packaging and bans on point of sale for tobacco, smoking in public places and cigarette vending machines. Therefore, stakeholders felt current policy in Scotland needs to focus on marketing and promotion of alcohol, as these have already been addressed in relation to tobacco:

*“Promotion and marketing is probably more important as a policy area at the moment for alcohol than tobacco. On the basis that a lot of the low hanging fruit for tobacco marketing has kind of been dealt with. And there are very few avenues for tobacco industries to market tobacco products. Whereas alcohol marketing is everywhere, it’s rife, particularly amongst children and young people. There are almost no limits.”* (S3, Third sector organisation)

Generally, stakeholders emphasized that policy success in tobacco can be used to advocate for change in alcohol control:

*“So if you think about the smoking ban in Scotland* [ban of smoking in indoor spaces introduced in 2006] *what it did was it de-normalised smoking in areas that people attend, you know? So imagine if you went to a supermarket that didn’t sell alcohol. It de-normalises that, “I’ve just walked up fifteen aisles and I got to the end one and, oh, there’s a bottle of wine for five ninety-nine, I’ll just chuck that in” you know?”* (S2, Third sector organisation)

*“There’s different things that we’re tackling with different aspects of it. Tobacco is looking at one aspect, alcohol is doing a different aspect. But it doesn’t mean that the two policy areas don’t learn from each other to see what could be implemented, and what we could learn from the wider world.”* (S10, Policy maker)

However, some emphasized that tobacco *“is a very different product to alcohol”* and *“therefore the policy approach is very different to it as well”* (S9, Policy)*:*

*“It’s [alcohol] to some extent a different kettle of fish, in the sense that the cultural elements of tobacco are different to the cultural elements of alcohol. Alcohol is significantly further behind in terms of how people view it as a health harming commodity, people generally view alcohol as fun, in the same way that, you know, tobacco was cool fifty years ago.”* (S3, Third sector organisation)

*“It’s much easier for tobacco in terms of harm from others, it’s much more direct and it’s harder with alcohol. It’s not just about the individual drinker, it’s the harm that it causes to families and communities.”* (S6, Public Health)

Given the relationship between availability, pricing and marketing, stakeholders discussed whether all three areas can be addressed together in one *“all-encompassing piece of legislation*”*:*

*“Availability also affects price. Price affects availability. Marketing affects availability. So in some ways having it all under one piece of legislation makes that relationship between them easier, because you can address them all at once”* (S8, Third sector organisation)

*“It’s a real interplay around different parts of policies around price, around availability, marketing, advertising, all of them, and how they all interlink. It’s not just a case of doing one thing, it’s how you address it across the piece.”* (S9, Policy maker)

Others believed *“we’ve got to be realistic about what we can achieve”* (S1, Third sector organisation) and suggested a *“stepped approach”* (S2, Third sector organisation) where different regulations are implemented at different stages. For example, one expert suggested that once a policy is implemented, it needs to be evaluated to *“demonstrate that it does have a positive impact on health outcomes”* (S8, Third sector organisation) and this evidence can then be used to advocate for subsequent policies.

### Policy priorities for addressing availability: Online availability as “a game changer”

Stakeholders started to unpack the notion of “availability,” suggesting that potential policies need to consider the different aspects of availability and how the retail landscape has changed over time. Some suggested that availability is more than just the number of shops:

*“Our thinking could be broader around availability… like for example with alcohol, and also with tobacco, our focus being on the number of premises, but actually how long they’re open for, how big their display size is, I think that there’s a lot of opportunities in there that we haven’t really tapped into. And probably we don’t want to, ‘cause the evidence is a little bit lacking.”* (S7, Public Health)

Participants talked about a shift in recent years toward increased purchasing of alcohol from off-sales, compared to on-sales premises^1^. This was believed to be accelerated by the pandemic when on-premises were closed, suggesting *“availability of alcohol in off-sales is probably going to be quite important to people.”* (S1, Third sector organisation).

Although we did not specifically ask about online availability, most respondents raised this issue, especially in relation to alcohol, and was described as *“a whole new dimension*” (S1, Third sector organisation) and a *“game changer”* (S7, Public Health). Stakeholders talked about a shift in recent years (especially since the Covid-19 pandemic) toward purchasing alcohol online, not only ordering via supermarkets or online retailers, but also via licensed premises and delivery companies. Some suggested that it contributed to increased accessibility of alcohol in rural areas:

*“I don’t think that living in a rural area has the same limited accessibility that it used to. You’ve got a lot more choice and access to a broader range of products. You might have to wait a little bit longer, compared to somebody in an urban centre. But not even that much longer, to be honest.”* (S7, Public Health)

According to some stakeholders, the current system for controlling alcohol availability is *“based on this idea that people go to their local shop and buy what they need”* (S7, Public Health), therefore failing to consider online availability. Some were concerned that restricting physical availability of alcohol (e.g., by reducing outlet density) could lead to an increase in online availability and suggested future legislation needs to account for this:

*“There is a risk that if you restrict availability in real life, then online life may see a proliferation. I think there needs to be something done potentially at the same time online.”* (S1, Third sector organisation)

Policy makers reported challenges around understanding and addressing online availability, including doorstep identification checks and online alcohol promotions for retailers not based in Scotland (where alcohol Minimum Unit Pricing has been implemented):

*“One issue that does give me a little bit of a headache is the challenge around online sales, because of the difficulties that are associated with that.”* (S11, Policy maker)

### Addressing alcohol and tobacco health inequalities: “you want the decisions to be taken at a local level”

Stakeholders believed that addressing health inequalities in relation to alcohol and tobacco requires the right political climate as *“all too often policy is driven by politics”* (S2, Third sector organisation):

*“The people who would have driven getting it to the legislative phase are no longer there, you have changes in government. The new Minister for Health comes in, kinda [kind of] goes, “Well, that’s yesterday’s problem” (…) that’s the political reality of the work.”* (S4, Third sector organisation).

One expert gave an example of this by saying that the implementation of Minimum Unit Pricing for alcohol in Scotland happened during a specific *“policy window”* when the new government were *“willing to be bold and different,” “didn’t have close links with the alcohol industry,”* and the issue of alcohol-related harm *“was very close to their hearts”* (S6, Public Health).

Considering the relationship between availability, pricing and marketing, stakeholders referred to restrictions in relation to reserved and devolved powers in the UK^2^, suggesting that availability is within the scope of the Scottish Government’s powers:

*“Out of marketing and price promotion, availability is probably the policy area that is wholly devolved to the Scottish Parliament.”* (S2, Third sector organisation)

Many suggested that any national approach needs to allow for local adaptation, as different areas (e.g., rural, deprived) experience different issues in relation to alcohol and tobacco availability:

*“You want the decisions to be taken at a local level because they should be responsive to local circumstances and local needs”* (S8, Third sector organisation)

Policy makers also stressed the importance of local approaches saying they *“try and respect at all times local democracy”* (S10, Policy maker). For example, some stakeholders suggested that rural communities experience different issues in relation to alcohol licensing:

*“One size doesn’t fit all. In some [rural] areas up north [of Scotland], alcohol would be what justifies and supports and sustains small local businesses, and enables them to survive. Probably more difficult to run that argument in Glasgow. [big city]”* (S14, Alcohol Licensing)

Stakeholders gave examples of essential services such as post offices and petrol stations, which were not *“economically viable”* in small rural communities and in order to continue to provide such essential services, *“they have to broaden out”* and apply for an alcohol license (S7, Public Health).

Stakeholders also reflected on alcohol and tobacco availability in areas of multiple deprivation. There was general acknowledgment that there are more outlets selling alcohol and tobacco in deprived communities than in affluent communities, creating an environment that drives people toward unhealthy choices:

*“We do have choice, but we also live in a world where our choices are heavily dictated to us quite often and if you live in an area of multiple deprivation and you have to walk past five fast food places and three places that are selling tobacco to get to the gym, you’re probably not gonna [going to] make it to the gym.”* (S2, Third sector organisation)

However, participants reflected that reducing the number of outlets selling unhealthy commodities requires a complicated decision-making process, which involves weighing the potential economic and wider public health benefits of an outlet against potential harms. This appeared to be particularly relevant to supermarkets, which sell both healthy and unhealthy products:

*“Somewhere like [deprived area] is crying out for a supermarket. (…) and I think you would probably struggle to find a supermarket that say they would open without an alcohol licence, because it is about providing the full service. (…) This is an opportunity to bring fresh fruit and veg, bakery provisions, at much more accessible pricing to an area that doesn’t currently have that service. There’s lots of positives, job creation, and it comes with alcohol. And it’s very difficult in those situations for the [licensing] Board to look at a ground for refusal when the benefits probably outweigh the potential for health-related harm.”* (S14, Alcohol Licensing)

Although stakeholders highlighted the benefits of policies that reflect the local context, they reported inconsistencies in *“approach, interpretation, implementation”* (S8, Third sector organisation) as a possible disadvantage. They gave an example of the current licensing system in Scotland where local Alcohol Licensing Boards can grant permissions for alcohol licenses. The Licensing Act (the Licensing [Scotland] Act 2005) that informs Alcohol Licensing Boards decisions includes a public health objective, which allows the Boards to refuse a license application which could undermine the objective to protect and improve public health. The stakeholders in our study reported inconsistencies in the use of the public health objective and issues around causality between availability and harm in different local areas, suggesting a need for clear guidance on how national policies can be consistently applied to reflect local needs.

When considering inequalities in relation to alcohol and tobacco-related harms, stakeholders suggested that the views of children and specific groups of the population that experience higher rates of alcohol-related harm need to be considered. Given measures have been implemented to reduce tobacco marketing in Scotland^3^, stakeholders focused on children’s exposure to alcohol. One suggestion was to reduce alcohol visibility in places where children go:

*“Kids that go there [local mixed-grocery shop] from the school to get their lunch and the way the queuing system works is you queue round the alcohol aisle (…) That seems a bit too much for me in terms of availability. It shows these kids where they can get it. They probably become familiar with brands.”* (S2, Third sector organisation).

Stakeholders talked about *“children’s rights to be protected from harms”* (S7, Public Health) saying that potential policy interventions can facilitate long-term change in children and young adults’ relationship with alcohol:

*“Children are the adults of the future, so how do we address how they look at alcohol? Because then that takes us through into how they are then approaching alcohol as adults. And into wider long-term use.”* (S9, Policy).

Others talked about restricting alcohol availability in venues frequented by children and young people. For example, one alcohol licensing expert reported an increase in applications for alcohol licenses from coffee shops. This participant felt such venues should be alcohol-free as children and young people often frequent coffee shops but reflected on the challenges of refusing an alcohol license and *“having to argue about the suitability of the premises and the activities, and it’s actually very, very difficult.”* (S14, Alcohol Licensing). Similarly, another expert expressed disapproval of occasional alcohol licenses for events, such as Christmas parties:

*“I cannot see why we should be issuing licensing where an event is predominately for children or young people. (…) An occasional license for an event, say a Christmas party for children, and they want an alcohol licence. We really don’t need to be supporting that.”* (S13, Alcohol Licensing).

Other suggestions to reduce children’s exposure and access to alcohol included banning alcohol sponsorships, not selling alcohol in venues with a soft play area, not selling alcohol in cinemas during specific hours and ID checks at alcohol aisles in supermarkets. One policy maker said it is important to engage with children:

*“One of the important things to recognise is taking the view of children into account, and listening to what children say themselves.”* (S9, Policy maker)

Stakeholders said that although structural policies can reduce children’s exposure to alcohol in their environment, *“you can’t legislate what people do inside their own homes”* (S3, Third sector organisation). They suggested mass media campaigns to educate parents about the harms of alcohol and tobacco, and challenge social norms around smoking and drinking (e.g., *“just raising awareness of children’s own experiences of exposure to alcohol,”* S8, Third sector organisation) in addition to the provision of targeted support (e.g., investment in smoking cessation services) to help people change their behavior:

*“If people’s parents smoke then they are more likely to smoke. And the counter to that is sort of two-fold. One, you can run mass media campaigns on dissuading people from smoking. And second, you can invest more in smoking cessation services and redirect people towards that. So if you help people’s parents stop smoking and their environment, the children are less likely to take up smoking ‘cause it’s not normal for them.”* (S3, Third sector organisation).

Third sector and public health stakeholders believed potential policies need to consider the experiences of people in recovery as *“temptation’s everywhere”* (S2, Third sector organisation):

*“If you’re in recovery, that can be quite a challenging experience when stuff pops up, when you’re not expecting it to. As a state, what responsibility do we have to people who want to make that choice, and who want to protect themselves? To me at the moment it feels too weighted to the side of the industry, and less so about people and people being able to say ‘I don’t wanna see that’.”* S7, Public Health

### Policy support and conflict of interest: “community involvement” and “open discussion”

We asked stakeholders about their views on the best ways to gain support among policy makers for alcohol and tobacco control policies. They talked about different factors that can influence policy, including the need for evidence and how it is communicated, and public support and engagement with the policy process. Policy makers stressed the importance of a *“a strong evidence base”:*

*“If we are looking at any policy changes at some future point, on what grounds? Where is the justification for change? What does the data that’s available actually tell us to help inform that policy-making decision?”* (S10, Policy).

A few stakeholders believed that *“policy makers are persuaded by public opinion”* (S8, Third sector organisation) so it is important to engage with the public to raise awareness of alcohol-related harm:

*“I think just raising awareness, being in the media, talking to people, that’s how policy makers are persuaded by public opinion. If you can win the public over then you’re on your way to a more successful policy approach, than if you’re just focusing on the policy makers themselves.”* (S8, Third sector organisation).

The combination of evidence and personal stories (*“people respond better to real life stories,”* S8, Third sector organisation) was believed to be particularly powerful. For example, one stakeholder talked about smoke-free spaces:

*“I think we probably do need community involvement, community voice in this as well. Because some of the most successful smoke-free playgrounds and schools have been where children have got involved and said, ‘please keep it away from us.’ And they’ve done posters and they’ve done awareness raising. And it’s come from their voice.”* (S5, Third sector organisation).

There was a common view among participants that retailers often represent the views of the alcohol industry and they should not be involved in policy formulation as they have *“too much of a vested interest”* (S4, Third sector organisation) and *“a lot of implicit power”* (S7, Public Health), which presents a conflict of interest:

*“Retailers are there to make a profit and they are part of the alcohol industry. My firm view is that no part of the alcohol industry should be a part of the policy formulation because it’s a complete conflict of interest”* (S4, Third sector organisation).

However, stakeholders believed it is important to hear retailers’ views in relation to feasibility and implementation of policy suggestions:

*“They* [retailers] *shouldn’t be involved in discussion about policy, in terms of setting policy. They have a role in terms of providing intelligence about the implementation of policy and things like that. They’ll know how it’ll work in practice, and they should be involved in those sorts of discussion. But not in terms of setting policy.”* (S6, Public Health).

*“There is a legitimate space for conversations with commercial interests when it’s about how proposed measures would impact on them and their business, and practicalities of making it work, but these are not health stakeholders and should not be allowed to influence or undermine health policy.”* (S5, Third sector organisation).

Policy makers said that involving retailers in policy discussions is *“a really complicated matter”* and depends on the specific policy and its context:

*“It could be different ministers, different aspects of it [the policy], because you’ve got the aspect of, ‘are you talking about from the point of view that are rural issues that you’re covering, or is it public health issues that you’re covering?’ So it really depends on the exact policy that you’re looking at.”* (S9, Policy maker).

A few stakeholders believed that the retail sector is not a *“monolith”* and supermarkets and smaller retailers have different needs, therefore there are *“different ways of engaging with them”* (S3, Third sector organisation):

*“I think there are genuine local retailers whose messages are not the same as the ones that their representatives sometimes are giving. And they need to be supported and listened to, and they’re part of their communities.”* (S5, Third sector organisation).

In terms of the best way to engage with retailers, expert suggested *“open discussion and dialogue”* (S5) in order to find *“common ground”* (S13, Alcohol Licensing):

*“Sometimes you can broadly want the same things… bigger supermarkets do have an interest in corporate responsibility and public health, they just need that sort of element of support and that feeling that we’re working with them, not against them.”* (S3, Third sector organisation).

## Discussion

This study provides insights from key stakeholders, based in Scotland, about alcohol and tobacco availability. This is one of the few studies to explore policy priorities for both commodities. Given that alcohol policies can influence smoking behaviors and vice versa, focusing on both commodities can facilitate strategic alignment across policy areas. Many of the stakeholders in our study indicated that alcohol and tobacco availability have not received as much policy attention from the Scottish Government as pricing and marketing of these products. Stakeholders highlighted the importance of public support and having sufficient evidence to inform policy. According to our participants key considerations for future policies include: drawing on lessons from tobacco control policies to address alcohol availability, considering different aspects of availability (especially online availability), ensuring policies reflect their local context, considering the impact of policies on children, and managing retailers’ involvement in the policy process.

Recognition that tobacco has distinct and particularly wide-ranging health harms has led to advances in tobacco control internationally and is known as ‘tobacco exceptionalism’ (Collin, 2012; McCambridge & Morris, 2019). Although tobacco is distinct in terms of health harms (half of all consumers of tobacco are killed by tobacco products), there are significant opportunities for greater policy coherence across unhealthy commodities. The stakeholders in our sample suggested that progress made in tobacco control provides important lessons for the prevention of alcohol-related harm. Although this may be feasible in relation to marketing and promotion, it may not be applicable in addressing alcohol availability. Alcohol can be purchased from a wide range of outlets within the on- and off-premise dichotomy, and can be consumed at the point of purchase (i.e., at on-premise outlets). There is a need for better understanding of how lessons from tobacco control can be applied to the wide diversity of alcohol outlet categories (e.g., pubs, restaurants, supermarkets, alcohol delivery services). Our participants spontaneously discussed online availability, especially of alcohol, and the importance of considering how restricting physical availability may lead to an increase in online availability. Policy makers reported challenges around understanding and addressing online availability and believed this needs to feature in policy discussions. Online alcohol delivery has been linked with heavier drinking (Huckle et al., 2021) and may facilitate the purchase of alcohol by underage drinkers (Mojica-Perez et al., 2019; Van Hoof et al., 2015). Online alcohol purchasing has been increasing internationally, and has been accelerated by COVID-19 lockdowns and broader switch to online retail (FARE, 2021; Grossman et al., 2022). Online availability needs to be considered as a key area in future policy interventions addressing alcohol availability. More research in the area is also needed to support policy interventions.

Stakeholders highlighted *“inter-dependencies”* between availability, marketing and price. Some suggested policies need to address all three, while others felt individual policies might be more feasible. However, there is little research exploring the relationships between place, availability and price (Holmes et al., 2014) limiting conclusions about what specific measures are needed to reduce harm. For example, pricing interventions may be effective in reducing availability of chaper alcohol in larger containers, often stocked by indepeent retailers in deprived areas (Ferguson et al., 2022; Forsyth et al., 2013). One example of successful implementation of indiviudal policies is in Lithuania where alcohol control policies have undergone a series of changes during the last three decades (Miščikienė et al., 2020). The first steps to restrict physical availability of alcohol were taken in 1995, with additional restrictions throughout the years, such as reduced hours for sale of alcohol at off-premises in 2018 and seasonal alcohol licenses ban in 2020 (Miščikienė et al., 2020). In comparison, Ireland recently implemented the Public Health (Alcohol) Act (Oireachtas, 2018), which addresses alcohol visibility, advertising and affordability (but not availability) in one all-encompassing piece of legislation. Despite industry lobbying, different measures within the Act have already been gradually implemented (e.g., Critchlow et al., 2022; Lesch & McCambridge, 2021). These examples illustrate the complexity of alcohol control policy development and implementation, and how policy may be influenced by political climate, lobbyist and public opinion.

The participants in our study argued that effective policy formulation and implementation require research evidence, public support and the right political climate. The stakeholders discussed examples of cultural change, such as when perceptions around smoking in indoor venues shifted following legislation, and de-normalised the behavior. A recent UK study found that there is public support for tobacco availability interventions, including requiring tobacco retailer licensing, restrictions on sales near schools and reducing the number of retailers selling tobacco in neighborhoods with a high density of tobacco retailers (Kock et al., 2022). According to the Overton Window model, policy makers are more likely to pursue policies that are widely accepted in society (and as such lie inside the Overton Window) (Mackinac Center for Public Policy, 2019). However, as public opinions change the ‘window’ can shift making policies more (or less) acceptable. The political climate is also important and stakeholders in our sample talked about government staff turnover and changing policy priorities. Smith (2021) refers to this as ‘institutional amnesia’ and notes that similar ideas may have to be re-presented as new ideas. The need for strong political leadership has previously been identified by tobacco control stakeholders in Scotland (Laird et al., 2019). They felt that future policies need to address the price and availability of tobacco products, and political leadership is important to sustain action and momentum on tobacco control (Laird et al., 2019).

Stakeholders in our sample indicated there is a need for a national approach to address alcohol and tobacco availability. They also stressed the importance of flexibility to adapt national policies to reflect the needs of local communities. The need for local policies might be particularly pronounced in rural and deprived areas. A scoping review of international literature suggests that hazardous alcohol use and alcohol-related harm may be higher in rural, compared to urban, areas (Friesen et al., 2022). Lack of recreational activities may contribute toward permissive social norms around drinking and drunkenness in rural areas (MacDiarmid, 2020). A recent WHO briefing (2022) highlights the importance of limiting alcohol outlets in areas of multiple deprivation to advance equity. In a modeling study, Caryl et al. (2019) found that a policy prohibiting tobacco sales in outlets that are over-represented in the most deprived areas had the potential to reduce inequalities between least and most deprived quintiles. Addressing health inequalities and the availability of alcohol and tobacco in areas of high deprivation needs to be central to public health strategies. This may require targeted approaches and public involvement to build shared understanding of alcohol problems within communities (Fitzgerald et al., 2017; Laird et al., 2019). However, research highlights the uncertainy around the effectiveness of public involvement in reducing alcohol availability (David et al., 2022). David et al. (2022) found that licensing authorities in England found it challenging to give weight to residents’ objectives due to lack of material evidence, do licensing authorities’ decisions were driven by a desire to achieve consensus, rather than the promotion of licensing objectives.

Local approaches to controlling alcohol availability can also target potentially sensitive locations, such as educational institutions and alcohol treatment facilities, and population groups who may be at the highest risk of experiencing harm, such as young people and people with alcohol use disorders (WHO, 2022). The stakeholders in our study talked about children’s exposure to alcohol products, which is particularly high in areas of multiple deprivation where there is higher outlet density (Hay et al., 2009; Macdonald et al., 2018; Shortt et al., 2015). For example, a GPS-tracking study demonstrated that children living in the most deprived areas in Scotland are five times more likely to be exposed to off-sales outlets than children from the least deprived areas (Caryl et al., 2022). Stakeholders believed restricting exposure to alcohol has potential to protect not only children but also people in recovery from alcohol dependence. Exploring policy options that are most feasible and effective in reducing exposure to alcohol and tobacco without exacerbating socioeconomic inequalities is a priority for future research (WHO, 2022; see also Caryl et al., 2019 for discussion on tobacco control policies; Shortt et al., 2018 on the role of the environment in alcohol recovery).

The stakeholders in our sample stressed the importance of policies to reflect local needs, but they also reported inconsistencies in policy implementation at local level, particularly in relation to the current alcohol licensing system in Scotland. This is in line with previous research in Scotland showing that licensing boards have capacity to shape the delivery of national policies by extending their scope or in some cases, causing them to be inconsistently applied (Fitzgerald et al., 2017, 2018). It also echoes the views of public health licensing stakeholders in England and Scotland who reported that in the current licensing system, alcohol availability may be contained but not reduced (O’Donnell et al., 2022). In some cases the public health values for reducing alcohol-related harms may be at odds with other council strategies (e.g., regenerate deprivered areas), and the perceived capacity to influence licensing decisions often influences public health practitioners’ action on individual licensing applications (Reynolds et al., 2018). This is not unique to the UK context. One study in Australia found that local government officers believed that national policies to limit alcohol outlet density may not be relevant to some local government areas and there is a need for more guidance on their implementation (Wilkinson et al., 2020). Inconsistencies in policy interpretation can impede local policy implementation (Abiona et al., 2019; Grace et al., 2016; Jankhotkaew et al., 2022; Kaewpramkusol et al., 2018; Trifonoff et al., 2014), so future policies to address health inequalities need to include clear guidance (Garthwaite et al., 2016).

Effective implementation of alcohol and tobacco control policies requires cooperation between different stakeholders, including government, retailers and the alcohol/tobacco industry. These stakeholders often have conflicting priorities. The alcohol industry, despite not being monolithic in terms of motives and power, has a commercial imperative to make a profit (Babor et al., 2023). This often competes with public health objectives, and can impede policy implementation. Our participants had different views on whether retailers represent the views of the alcohol industry and whether they should be involved in alcohol policy discussions. A few believed that involving retailers in policy discussions represents a conflict of interest and can impede effective policies. Others suggested that retailers need to be involved in discussions regarding policy feasibility (but not policy formulation) as they may be responsible for implementation. Policy makers need to be able to consider competing ideas in each policy area, with a focus on exploring what key actors believe to be in their interest and why (Smith, 2021). Policy framings are important influencing policy makers’ decisions (Katikireddi et al., 2014; Nicholls & Greenway, 2015; Thom et al., 2016). For example, Katikireddi et al. (2014) show that a change in policy debate’s framing was important for the implementation of Minimum Unit Pricing for alcohol in Scotland, as industry and public health actors adopted different framings when presenting research evidence. Industry-related groups framed alcohol problems in narrow terms, while non-industry actors characterized alcohol as a policy issue that adversely affected the whole Scottish society (Katikireddi et al., 2014). Nicholls & Greenway (2015) discuss the complex interplay of alcohol problem definitions, political ideology, systemic tensions between government departments and different bodies of evidence, in developments (or lack of) in alcohol policy. Often, public health advocates need to influence policy by framing arguments around alcohol-related harms in a way that can convince policy makers that action is needed (Thom et al., 2016).

## Strengths and limitations

This is one of the few studies exploring stakeholders’ views on alcohol and tobacco availability. Although participants’ views are grounded in their experience, primarily in the Scottish policy context, they are relevant to other contexts and countries. The sampling for this study reflects a bias toward public health stakeholders, some of whom have been involved in alcohol and tobacco policy advocacy. We were unable to recruit alcohol and tobacco retailers who may have offered different views on alcohol and tobacco control policies (e.g., Burton et al., 2018). Another limitation of the current study is that discussion focused primarily on spatial availability of alcohol (and not temporal), and we didn’t conceptualize spatial availability (e.g., outlets per population size, outlets per geographical area). Stakeholders also discussed availability in terms of aggregated categories (i.e., off-premise and on-premise outlets). This lack of specificity limits conclusions about policy suggestions to address the different dimensions of alcohol availability. The importance of understanding the relationship between different types of alcohol availability and outlets, and related harms has been discussed in a review of international literature (Holmes et al., 2014).

## Conclusion

Discussions with alcohol and tobacco stakeholders from third sector organizations, government, public health and alcohol licensing highlighted key considerations that can be used to inform future policies to address alcohol and tobacco availability. These include drawing on lessons from tobacco control policies, considering different aspects of availability (especially online availability), ensuring policies reflect the local context they are implemented in, considering the impact of policies on children, and managing retailers’ involvement in the policy process.

## Supplementary Material

Supplemental Material

## Notes

1 Alcohol outlets include on-premise (i.e. places that sell alcohol products that are meant for consumption while visiting the place, such as a pub, bar or restaurant) and off-premise (i.e. places like convenience stores and supermarkets or alcohol-only shops that sell alcohol products, meant for consumption elsewhere)

2 In the United Kingdom (UK), the Scottish Government has devolved powers meaning it can introduce some policies independently of the UK government. The UK Parliament and UK Government retain some powers across the whole of the UK and these are referred to reserved powers.

3 Some policies include the Health Act 2009 banning the display of tobacco products at the point of sale in supermarkets from 2013 and smaller retailers from 2015, ASH, 2013; The United Kingdom Standardized Packaging of Tobacco Products Regulations 2015 and the Tobacco and Related Products Regulations 2016 mandating standardised tobacco packaging, Critchlow et al., 2022
