# Supplementary material for: “Availability is the poor cousin of marketing and pricing”: qualitative study of stakeholders’ views on policy priorities around tobacco and alcohol availability
Source: Drugs (Abingdon Engl). 2023 Nov 25;32(1):51–62. doi: 10.1080/09687637.2023.2282355 (PMC11771472; doi:10.1080/09687637.2023.2282355)
Supplement: Supplemental Material [file IDEP_A_2282355_SM7163.docx]

**Alcohol and tobacco availability in neighbourhoods across Scotland**

Interview topic guide

| **Note on using this topic guide:**  We will conduct individual interviews with stakeholders to explore relevant topics in line with our research questions as well as remain alert to additional topics raised by the participants. Questions and prompts will be tailored to each participant.   - The interviews may be conducted face-to-face or online. We will follow current government advice in relation to COVID-19 at the time of data collection. - This topic guide has been designed to be used flexibly. This means that the question wording, order in which issues are covered and the time spent on different topics will vary between interviews. - We may raise related issues not outlined below as they arise in the course of the data collection, providing that to do so raises no ethical issues or risks to participants not already outlined in the Participant Information Sheet. - Prompts may be used by the researcher. Participants can discuss a topic spontaneously before prompting specific issues. There is no expectation that all prompts are covered for any one question in the list of topics or in every interview. |
| --- |

**Welcome and introduction**

- Explain purpose of study
- Explain how data will be audio-recorded, stored, transcribed, anonymised, published etc. (e.g. all personal identifiable information removed). Explain confidentiality.
- Request informed consent
- Emphasise there are no ‘right or wrong’ answers – interested in the respondent’s experience. Ask if any questions about the study.

**QUESTIONS FOR POLICY MAKERS AND THIRD SECTOR ORGANISATIONS**

- Please can you tell me about your job and how long you have been in this role?
- Tell me about the work your organisation does in the alcohol/tobacco field?
- What are your views on alcohol/tobacco availability in Scotland? *Prompt about views on perceived importance of addressing availability*
- How important is availability in the current policy landscape compared to price and promotion / marketing?
- Do you think there are gaps in current alcohol/tobacco policies in Scotland?
- If given the opportunity, what is the first thing you would do to reduce alcohol / tobacco availability?

Use recent relevant policy documents from other countries (e.g. For tobacco: “Proposals for a Smokefree Aotearo 2025 Action Plan”) and discuss in relation to Scotland.

Present list of policies to tackle alcohol availability (e.g. alcohol-free spaces, restricting hours and days of alcohol sale, regulate number, density and / or location of retail alcohol outlets) or tobacco availability (e.g. tobacco free spaces for children, cap number of retailers or require minimum spacing between tobacco retailers to prevent clustering). Prompt discussion and focus on feasibility, barriers, potential perceived success of each intervention.

- Should the retail sector be involved in discussions about reducing alcohol / tobacco harm and if so, how?
- How do you build support for alcohol / tobacco policies from the public and key stakeholders? What approaches have you found particularly useful?

**QUESTIONS FOR RETAILERS**

- Please can you tell me about your job and how long you have been in this role?Tell me about your premise(s) and what area it serves? Are there similar shops nearby? How do you attract customers?
- Ask about whether they are particularly interested in attracting a certain group of customers.
- Ask about competition between retailers (e.g. low prices, deals).
- Ask about opening times and if they are different on different days of the week and why.
- What is the relationship between your premises and the community / communities they serve? *(shape question accordingly depending on whether retail stakeholder is from a local shop or a big retailer at multiple locations)*
- What are your views about current Scottish Government policies (e.g. ban of tobacco point of sale displays, MUP etc.)? *Specifically focus on alcohol / tobacco availability.*

Present list of policies and discuss focusing on feasibility, barriers, potential perceived success of each intervention. (as above)

- How can we balance the needs of the retail sector versus the need to protect the health of the public in Scotland? Is this a conversation that retailers should be involved in?
- Should the retail sector be involved in discussions about reducing alcohol / tobacco harm and if so, how?

**Final comments and debrief**

- Any final comments?
- Repeat the purpose of the study and explain when findings will be available.
- Thank the participants for their time and input.
